# Effects and plasma proteomic analysis of GLP-1RA versus CPA/EE, in combination with metformin, on overweight PCOS women: a randomized controlled trial

**DOI:** 10.1007/s12020-023-03487-4

**Published:** 2023-08-31

**Authors:** Mingyu Liao, Xing Li, Hao Zhang, Ling Zhou, Liu Shi, Weixin Li, Rufei Shen, Guiliang Peng, Huan Zhao, Jiaqing Shao, Xiujie Wang, Zheng Sun, Hongting Zheng, Min Long

**Affiliations:** 1 Department of Endocrinology, Translational Research Key Laboratory for Diabetes, Xinqiao Hospital, Army Medical University, Chongqing, 400037 China; 2 Department of Endocrinology, Jinling Hospital, Affiliated Hospital of Medical School, Nanjing University, Nanjing, 210016 China; 3 Key Laboratory of Genetic Network Biology, Collaborative Innovation Center of Genetics and Development, Institute of Genetics and Developmental Biology, Chinese Academy of Sciences, 100101 Beijing, China; 4 Univeristy of Chinese Academy of Sciences, 100049 Beijing, China; 5 Department of Endocrinology, Southwest Hospital, Army Medical University (Third Military Medical University), Chongqing, 400038 China; 6 Department of Medicine, Division of Diabetes, Endocrinology and Metabolism, Baylor College of Medicine, Houston, TX USA

**Keywords:** PCOS (Polycystic Ovary Syndrome), Glucagon-like Peptide-1 Receptor Agonist (GLP-1 RA), Metformin, Proteomics Analysis

## Abstract

**Purpose:**

Polycystic ovary syndrome (PCOS) is characterized by reproductive dysfunctions and metabolic disorders. This study aims to compare the therapeutic effectiveness of glucagon-like peptide-1 receptor agonist (GLP-1RA) + Metformin (Met) versus cyproterone acetate/ethinylestradiol (CPA/EE) + Met in overweight PCOS women and identify potential proteomic biomarkers of disease risk in women with PCOS.

**Methods:**

In this prospective, open-label randomized controlled trial, we recruited 60 overweight PCOS women into two groups at a 1:1 ratio to receive CPA/EE (2 mg/day: 2 mg cyproterone acetate and 35-μg ethinylestradiol,) +Met (1500 mg/day) or GLP-1 RA (liraglutide, 1.2–1.8 mg/day) +Met (1500 mg/day) for 12 weeks. The clinical effectiveness and adverse effects were evaluated, followed by plasma proteomic analysis and verification of critical biomarkers by ELISA.

**Results:**

Eighty(80%) patients completed the study. Both interventions improved menstrual cycle, polycystic ovaries, LH(luteinizing hormone) and HbA1c(hemoglobin A1c) levels after the 12-week treatment. GLP-1RA + Met was more effective than CPA/EE + Met in reducing body weight, BMI (Body Mass Index), and waist circumference, FBG(fasting blood glucose), AUCI(area under curve of insulin),TC (Total Cholesterol), IL-6(Interleukin-6) and improving insulin sensitivity, and ovulation in overweight women with PCOS, with acceptable short-term side effects. CPA/EE + Met was more effective in improving hyperandrogenemia, including T(total testosterone), LH, LH/FSH(Luteinizing hormone/follicle-stimulating hormone), SHBG(sex hormone-binding globulin) and FAI (free androgen index). By contract, GLP-1RA+Met group only improved LH. Plasma proteomic analysis revealed that the interventions altered proteins involved in reactive oxygen species detoxification (PRDX6, GSTO1, GSTP1, GSTM2), platelet degranulation (FN1), and the immune response (SERPINB9).

**Conclusions:**

Both CPA/EE+Met and GLP-1RA + Met treatment improved reproductive functions in overweight PCOS women. GLP-1RA + Met was more effective than CPA/EE + Met in reducing body weight, BMI, and waist, and improving metabolism, and ovulation in overweight women with PCOS, with acceptable short-term side effects. CPA/EE + Met was more effective in reducing hyperandrogenemia. The novel plasma biomarkers PRDX6, FN1, and SERPINB9, might be indicators and targets for PCOS treatment.

**Trial registration ClinicalTials.gov Trial No:**

NCT03151005. Registered 12 May, 2017, https://clinicaltrials.gov/ct2/show/NCT03151005.

## Introduction

Polycystic ovary syndrome (PCOS) is a common cause of female reproductive dysfunction [1]. Classic PCOS is defined by the diagnostic criteria of the National Institutes of Health as hyperandrogenism and ovulatory dysfunctions, and affects 6–10% of women of reproductive age [2]. However, the prevalence may be twice as high under the broader Rotterdam criteria (hyperandrogenemia, ovulatory dysfunction, or polycystic changes [3]). Over 50% of women with PCOS are overweight or obese [4, 5] with insulin resistance [6]. Obesity and hyperinsulinemia also contribute to the pathogenesis of PCOS [7]. Treatment for PCOS is symptom-oriented, with pharmacological treatments targeting androgen excess, oligo-ovulation, and insulin resistance [8].

Currently, the pharmacological therapy of PCOS includes oral contraceptives, insulin sensitizers, weight-loss drugs, and ovulation induction agents. A number of monotherapies have been used to treat overweight PCOS patients, but it is hard to improve both hyperandrogenism and metabolic disorders. Hormonal contraceptives, such as co-cyprindiol (cyproterone acetate/ethinylestradiol, CPA/EE) consisting of 2 mg cyproterone acetate (CPA) and 35 μg ethinylestradiol (EE), are recommended for the management of PCOS patients with hyperandrogenism [9] to manage menstruation regularity, reduce androgen levels, and improve hairiness. However, CPA/EE has limited effectiveness in managing metabolic disorders, such as hyperglycemia, dyslipidemia, and insulin resistance, especially in overweight/obese patients.

Metformin is widely used to treat hyperglycemia, hyperinsulinism, and even hyperandrogenism through improving glucose uptake and insulin sensitivity [10]. In recent years, emerging glucagon-like peptide-1 receptor agonists (GLP-1RAs) show favorable effects on metabolism. Several studies demonstrated that the administration of GLP-1RAs reduces body weight and improves metabolic parameters in obese women with PCOS [11]. GLP-1RAs act as effective insulin sensitizers and can improve reproductive outcomes through weight loss [12]. These advantages suggest that GLP-1RAs is useful for treating PCOS in overweight patients.

However, there are few reports on the effects of GLP-1RA combined with metformin on glucose and lipid metabolism and reproductive function in PCOS patients. We hypothesized that the GLP-1RA +Met has better efficacy in reducing weight loss and improving glucose and lipid metabolism and reproduction in PCOS patients. Thus, we evaluated the efficacy of GLP-1RA + Met in the treatment of weight, metabolic and endocrine parameters in overweight women with polycystic ovary syndrome through clinical trials, and we further performed plasma proteomics profiling to explore potential mechanisms and to identify biomarkers of therapeutic effectiveness. Finally, We present the following article in accordance with the CONSORT reporting checklist.

## Materials and methods

### Study population

**Fig. 1:**
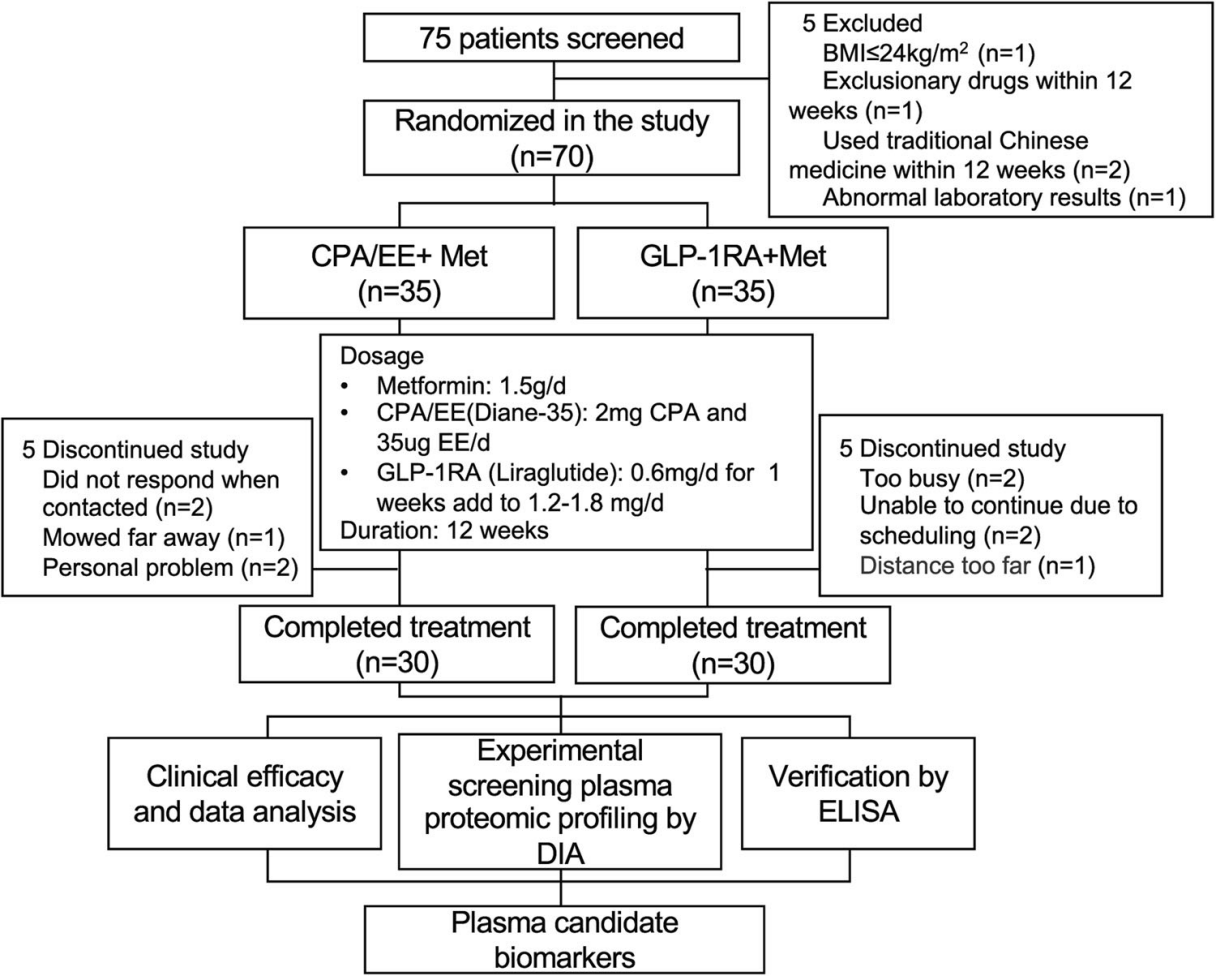
Study flowchart. PCOS polycystic ovary syndrome, GLP-1RA glucagon-like peptide-1 receptor agonist, CPA cyproterone acetate, EE ethinylestradiol

The clinical trial ran from July 11th 2017 to April 31st 2021. Seventy-five patients diagnosed with overweight or obese [13] and PCOS were recruited from the Endocrinology Department of the Second Affiliated Hospital, Army Medical University, China. Among them, 70 women matched the inclusion criteria. A total of 10 participants were excluded or dropped out from further analyses, and the remaining 60 patients completed the study. The study was completed by two groups, namely the CPA/EE + Met group (30 cases) and the GLP-1RA + Met group (30 cases). The participants of the study were randomly divided into these two groups using a table of random numbers (Fig. 1). The study was approved by the ethics committee of Xinqiao Hospital and Clinical trial registration (No. NCT03151005). All subjects were given a detailed description of the purpose, method, and significance of the experiment before informed consent was obtained.

### Inclusion and exclusion criteria

The patient inclusion criteria were (a) clinical diagnosis of PCOS according to the Rotterdam criteria based on the presence of two of three criteria (3), oligomenorrhoea, clinical or biochemical hyperandrogenism, and polycystic ovaries on ultrasound after exclusion of other endocrine causes of hyperandrogenism; (b) participants had no concurrent illness and were not on any prescription or over-the-counter medication that was likely to affect insulin sensitivity or lipids for the preceding 12 weeks; (c) participants were advised not to change physical activity or dietary habits during the study period; (d) given that all subjects were of Asian ethnicity, overweight was defined as BMI ≥ 24 kg/m^2^ [13]. (e) all subjects had normal thyroid-stimulating hormone and prolactin levels.

The patient exclusion criteria were (a) age below 18 years or over 50 years; (b) uncontrolled hypertension (blood pressure ≥160/100 mmHg); (c) signs of liver or renal failure or active liver disease (alanine transaminase (ALT) > 2.5× the upper limit of normal values); (d) patients who were postmenopausal or pregnant; (e) alcohol intake greater than 20 g/day; (f) patients who could not complete the intervention or had other conditions that made them ineligible for participation. For example, patients taking glucocorticoid steroids or under treatment for a malignant tumor were excluded.

### Methods and interventions

Each participant in the CPA/EE + Met group took 0.5 g metformin 3 times per day and one tablet of CPA/EE consisting of 2 mg cyproterone acetate and 35-μg ethinylestradiol, per day (starting on the first day of each menstrual cycle for 21 consecutive days and then pausing for 7 days) for 12 weeks. Each participant in the GLP-1RA + Met group took 0.5 g metformin 3 times per day, liraglutide was administered subcutaneously at a dose of 0.6 mg once daily and increased to 1.2 mg/day after one week, with a maximum dose of 1.8 mg/day, Pregnancy is not allowed throughout the treatment.

### Observation indexes

General clinical data, such as height, weight, waist circumference, hip circumference, and Ferriman-Gallwey score, were collected at baseline and post-treatment for calculating body mass index (BMI) and waist-hip ratio (WHR). Information on menstrual cycles was recorded.

Biochemical measurements: Plasma and serum were harvested for biochemical tests. Blood samples were collected while the participants were in a fasted state on the 3rd to 5th day of the menstrual cycle or during the follicular stage. The fasting blood glucose (FBG) level was measured by the glucose oxidase method, and glycosylated hemoglobin (HbA1c) was assessed by anion-exchange high-performance liquid chromatography. The fasting serum insulin (FINS) level was measured by radioimmunoassay. An oral glucose tolerance test (OGTT) and insulin release test were conducted for all PCOS subjects. Each participant ingested 75 g glucose in 5 min, and we measured the blood insulin level before the start of the test (0 min) and at 30, 60, 120, and 180 min. The homeostasis model was used to evaluate the insulin resistance index (HOMA-IR = FINS×FBG/22.5) and pancreatic β-cell function [HOMA-β = FINS × 20/(FBG-3.5)]. Blood lipid mass spectrometry, including triglyceride (TG), total cholesterol (TC), high-density lipoprotein cholesterol (HDL-C), and low-density lipoprotein cholesterol (LDL-C), was detected by an automatic biochemical analyzer. Levels of testosterone (T), follicle-stimulating hormone (FSH), and luteinizing hormone (LH) were measured by an IMMULITE 2000 Immunoassay System, and the LH-FSH ratio was calculated. Serum sex hormone-binding globulin (SHBG) and dehydroepiandrosterone (DHEA-S) levels were assessed by immunoradiometric assays. All the above indicators were measured before and after treatment. Experimental normal ranges and kit suppliers are listed in Supplementary Table 2.

The primary outcomes were changes in reproductive hormone levels along with glucose and lipid metabolism associated with obesity. The secondary outcomes included anthropometric changes as well as plasma proteomics analyses related to PCOS. Throughout the study, adverse events were recorded through direct questioning, patient self-reporting, physical examination, and clinical laboratory tests.

### Plasma sample collection and extraction for proteomic analysis and ELISA

For further proteomic analysis and ELISA detection, plasma was collected into EDTA tubes, centrifuged for 15 min at 1000 × *g* at 2–8 °C within 30 min of collection, and stored at −80 °C. For proteomic analysis, proteins were extracted from each 100 μL EDTA-treated plasma sample, followed by reduction with dithiothreitol, alkylation with iodoacetamide, and overnight digestion at 37 °C with trypsin (Hualishi Tech. Ltd, Beijing, China).

### Proteomic data acquirement and analysis

**Fig. 2:**
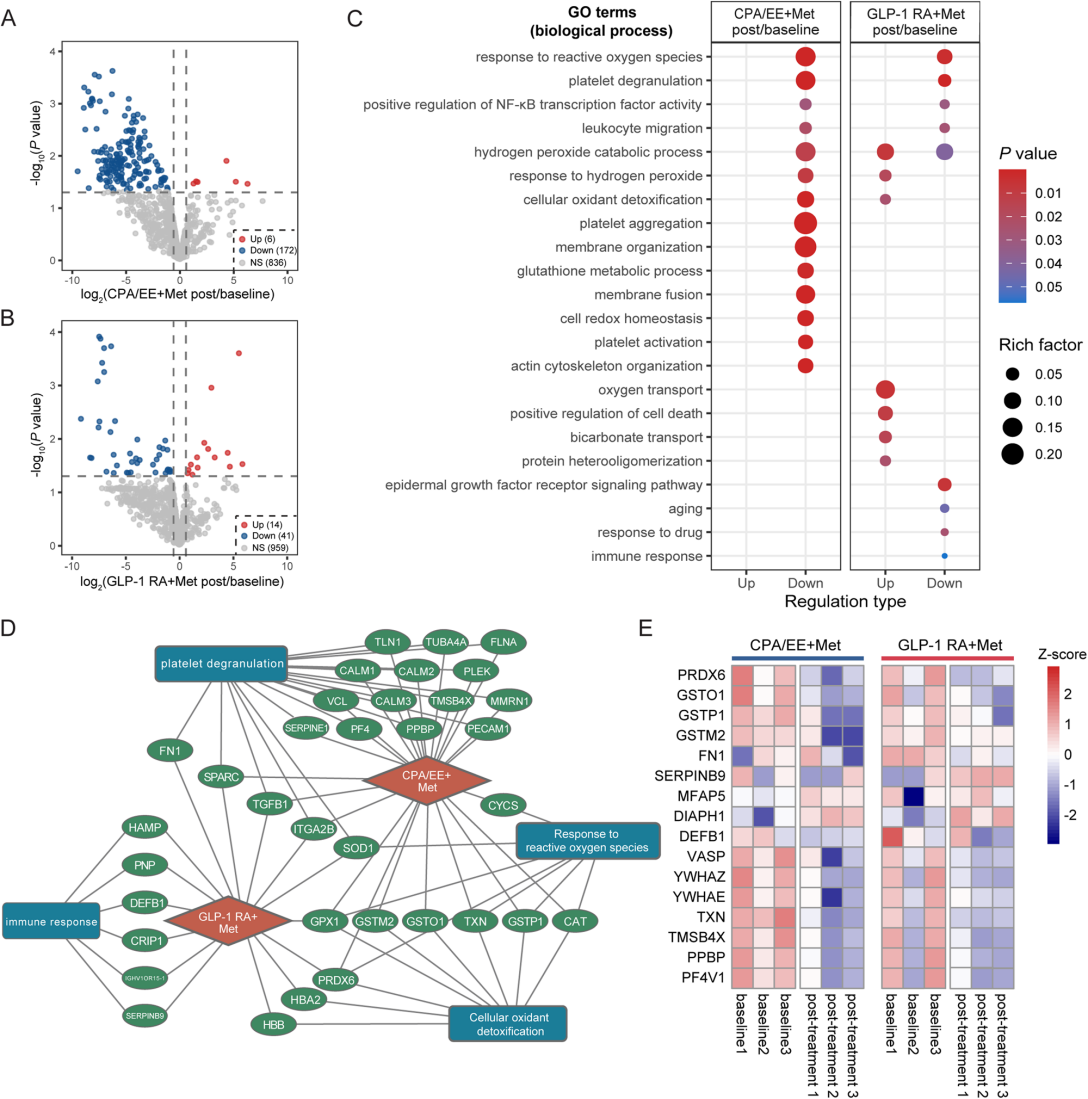
Proteomic analysis of plasma from PCOS pa tients treated with CPA/EE+Met or GLP-1RA+Met. Volcano plots of differentially expressed plasma proteins from patients prior to or after treatment with CPA/EE+Met (**A**) or GLP-1RA+Met (**B**). Red and blue dots represent significantly up- or downregulated protein expression, respectively (fold change ≥1.5 and *P* value < 0.05, moderated paired *t* test by limma, *n* = 3). Gray dots show nonsignificantly changed proteins. (**C**) Gene ontology (biological process) enrichment analysis of differentially expressed proteins from the two groups. The rich factor was calculated by the number of proteins enriched in a specific item divided by the total number of proteins in that term. (**D**) Network showing interactions between drugs (orange diamonds), differentially expressed proteins (green circles), and selected enriched functional terms (blue squares). (**E**) Abundance heatmap of sixteen selected proteins prior to and after treatment

Plasma protein mass spectrometry analysis was conducted in 3 patients in each group. Specifically, 20 μg protein was separated into peptide fractions by an LC-20AB HPLC system (Shimadzu, Kyoto, Japan). Peptide fractions were loaded onto a Thermo UltiMate 3000 UHPLC system coupled with a Q-Exactive HF mass spectrometer (Thermo Fisher Scientific, San Jose, CA, USA) for peptide separation and identification. A proteome spectral library was built in data-dependent acquisition (DDA) mode, followed by peptide identification in data-independent acquisition (DIA) mode using a sequential window acquisition of all theoretical fragments (SWATH) method. In DDA mode, the top 20 precursors with charge statuses ranging from +2 to +6 were selected for fragmentation during the MS2 scan. For spectral data analysis, peptide identification was conducted using MaxQuant ver. 1.5.3.30 against the UniProtKB/Swiss-Prot protein knowledgebase (Release 2019_06, https://www.uniprot.org/), followed by spectral library construction using Spectronaut™ (ver. 12, Biognosys, Schlieren, Switzerland). DIA data corrected for retention time by indexed retention time (iRT) were sent for peptide quantification in Spectronaut™. Next, the peptide quantification data were preprocessed using MSstats ver. 3.20.1 [14]. In short, peptide intensities were normalized by equalizing intensity medians across samples. The top 3 matching peptides with the highest average of log_2_ intensity of each protein were used to generate protein-level abundance data. Proteins with missing values in more than 50% of samples were removed and missing values of proteins were replaced with the minimum of all samples. After preprocessing, Pearson correlation analysis was conducted across samples using the “cor” function in the R package stats ver. 4.0.2 and visualized with corrplot ver. 0.84. Differentially expressed proteins were identified using limma ver. 3.44.3. [15] in paired mode under the condition of fold change ≥ 1.5 and *p* value < 0.05(moderated paired *t* test by limma). Gene ontology enrichment analysis was performed using Database for Annotation, Visualization, and Integrated Discovery (DAVID) ver. 6.8 [16] with UniProt Entry ID as input and visualized using ggplot2 ver. 3.3.2. A protein expression heatmap was built using pheatmap ver. 1.0.12 from the R library. A drug-protein-functional term network was constructed using Cytoscape ver. 3.8.1. [17]. Correspondingly screened proteins were used to construct a drug-protein-functional term network (Fig. 2D). Six protein candidates representing response to reactive oxygen species and cellular oxidant detoxification (PRDX6, GSTO1, GSTP1, GSTM2), platelet degranulation (FN1), and immune response (SERPINB9) were selected for further experimental validation (Fig. 2E, Supplementary Fig. 2B).

### ELISA validation

The validation analysis of the differential plasma proteins was performed by ELISA, following the manuals of corresponding ELISA kits for GSTP1 (ELH-GSTP1-1, Raybiotech, Georgia, USA), GSTM2 (P81730, Cusabio, Wuhan, China), GSTO1 (Abbexa, abx151761, TX 77042, USA), PRDX6 (SEF756Hu, Cloudclone, Wuhan, China), Fibronectin (FN1) (ab108848, Abcam, Cambridge, USA), and SERPINB9 (SED390Hu, Cloudclone, Wuhan, China).

### Statistical analysis

Statistical analyses were performed using R ver. 4.0.2, SPSS ver. 23.0 and GraphPad Prism 8. The sample size was calculated using PASS software, with means and variances set according to our preliminary experimental results. Power was set at 0.8, and significance level at 0.05. All continuous variables were described as the mean ± standard deviation (SD), both at baseline and post-treatment, for each group. Two-way ANOVA was used for analysis. Pearson χ2 tests or Fisher exact tests were used for the analysis of classified data. The nonparametric Mann–Whitney U test was utilized to compare changes in clinical parameters between different treatment groups. Proteomic data acquisition and analysis were described. Protein levels were described as the mean ± standard error of the mean (SEM), and the Student’s paired *t*-test was used to compare plasma protein levels measured by ELISA. *P* values < 0.05 were considered statistically significant.

## Results

### Clinical characteristics of the cohort

The two groups were well matched at baseline. The thorough assessment of clinical parameters before and after treatment in each group is shown in Table 1. These parameters encompassed basic biometric features (weight, BMI, WHR, reproductive function, F-G score, percentage with dominant follicles, regular menstruation, amenorrhea, oligomenorrhea, and polycystic ovaries according to Rotterdam criteria, sex-related hormones), biochemical profile of glucose metabolism (HbA1c, FBG, AUC-insulin, OGTT-insulin, and HOMA-IR), lipid profiles, liver/kidney function, and inflammation status. The adverse effects are listed in Supplementary Table 1. Weight gain, irregular menstrual bleeding, and elevated glutamic-pyruvic transaminase levels occurred in 13.33, 6.67, and 3.33% of patients, respectively, after CPA/EE + Met treatment. After GLP-1RA+Met treatment, diarrhea, nausea, vomiting, and indigestion occurred in 13.33, 13.33, 13.33, and 6.67% of patients, respectively (Supplementary Table 1).

**Table 1:** Baseline and post-treatment characteristics of clinical parameters after 12-week of treatment

	CPA/EE+Met (*n* = 30)			GLP-1 RA+Met (*n* = 30)		
Characteristics	Baseline	Post-treatment	*P* value	Baseline	Post-treatment	*P* value
Biometric features						
Weight (kg)	71.14 ± 10.99	69.29 ± 10.43	0.497	74.56 ± 10.20	67.16 ± 10.40^**^	0.008
BMI (kg/m²)	27.75 ± 4.47	26.99 ± 4.04	0.452	29.23 ± 3.34	26.32 ± 3.67^**^	0.005
Waist (cm)	93.07 ± 9.66	90.53 ± 9.94	0.315	95.78 ± 9.59	88.57 ± 9.78^**^	0.005
WHR	0.90 ± 0.06	0.89 ± 0.07	0.553	0.91 ± 0.06	0.89 ± 0.07	0.237
SBP (mm/Hg)	121.43 ± 13.82	122.17 ± 6.35	0.994	119.30 ± 14.41	122.27 ± 10.07	0.775
DBP (mm/Hg)	75.97 ± 6.87	76.27 ± 7.05	0.888	79.60 ± 8.76	79.70 ± 9.74	0.962
Reproductive function						
Dominant follicle, n (%)	0 (0.00)	1 (3.40)	1.000	0 (0.00)	6 (20.00)^*^	0.031
Regular menstruation, n (%)	7 (23.33)	23 (76.66)^***^	<0.001	8 (26.67)	22 (73.33)^***^	<0.001
Amenorrhea, n (%)^a^	12 (40.00)	0 (0.00)^***^	<0.001	9 (30.00)	2 (6.67)^*^	0.020
Oligomenorrhoea, n (%)^b^	11 (36.67)	8 (26.67)	0.405	13 (43.33)	6 (20.00)	0.052
Polycystic ovaries according to Rotterdam criteria, n (%)^c^	12 (40.00)	0 (0.00)^***^	<0.001	9 (30.00)	2 (6.67)^*^	0.020
F-G score	2.97 ± 2.26	2.43 ± 1.88	0.266	3.23 ± 2.19	2.87 ± 1.90	0.419
T (nmol/L)	2.59 ± 1.08	1.82 ± 0.51^***^	0.002	2.36 ± 1.00	2.14 ± 1.02	0.362
LH (mIU/ml)	9.93 ± 5.69	5.64 ± 2.27^***^	<0.001	8.41 ± 4.62	5.45 ± 3.42^**^	0.008
FSH (mIU/ml)	4.38 ± 1.53	4.41 ± 1.45	0.936	4.32 ± 1.72	3.82 ± 1.07	0.188
LH/FSH	2.30 ± 1.06	1.42 ± 0.82^**^	0.001	2.08 ± 1.28	1.48 ± 0.83	0.023
DHEA-S (ug/dL)	400.46 ± 154.78	397.77 ± 118.14	0.951	393.52 ± 204.04	360.18 ± 188.02	0.447
SHBG (nmol/L)	40.53 ± 38.60	72.80 ± 62.44^**^	0.003	35.42 ± 36.39	30.40±21.15^**^	0.646
FAI	12.03 ± 9.82	5.47 ± 5.58*^*^	0.002	10.60 ± 6.48	11.60 ± 10.46	0.087
E2 (pg/ml)	65.73 ± 34.22	61.53 ± 42.65	0.698	66.93 ± 47.84	65.63 ± 41.43	0.999
P (ng/ml)	0.63 ± 1.02	0.73 ± 2.23	0.698	1.10 ± 2.33	0.89 ± 1.63	0.904
PRL (ng/ml)	18.83 ± 11.27	15.29 ± 8.90	0.265	15.95 ± 7.21	15.68 ± 18.48	0.932
Biochemical profile of glucose metabolism						
HbA1c (%)	5.57 ± 0.37	5.14 ± 0.33^***^	<0.001	5.85 ± 0.55	5.18 ± 0.37^***^	<0.001
HbA1c (mmol/mol)	37.43 ±4.06	32.57 ± 3.75^***^	<0.001	40.47 ± 5.96	33.07 ± 4.04^***^	<0.001
FBG (mmol/L)	4.93 ± 0.51	4.56 ± 0.54	0.067	5.39 ± 1.14	4.85 ± 0.76^**^	0.008
AUCI	288.90 ± 151.29	262.88 ± 196.53	0.908	280.79 ± 141.45	182.47 ± 96.72^***^	<0.001
FINS (uU/ml)	15.55 ± 6.25	15.76 ± 10.69	0.922	19.10 ± 10.01	12.89 ± 4.50^**^	0.004
OGTT-30 min insulin(uU/ml)	103.66 ± 44.40	89.29 ± 45.41	0.333	103.21 ± 74.73	74.14 ± 59.02	0.052
OGTT-60 min insulin(uU/ml)	113.65 ± 67.63	105.92 ± 66.66	0.610	111.92 ± 57.96	75.19 ± 36.83*	0.017
OGTT-120 min insulin(uU/ml)	114.34 ± 66.40	103.93 ± 97.95	0.591	111.59 ± 75.10	70.76 ± 52.48*	0.037
OGTT-180mininsulin(uU/ml)	67.21 ± 67.87	62.54 ± 74.00	0.749	57.75 ±45.20	30.04 ± 26.33*	0.041
HOMA-IR	3.45 ± 1.54	3.27 ± 2.46	0.723	4.57 ± 2.40	2.81 ± 1.12^***^	<0.001
Biochemical profile of lipid metabolism						
TG (mmol/L)	1.68 ± 1.06	1.70 ± 0.77	0.933	2.01 ± 0.88	1.66 ± 0.99	0.148
TC (mmol/L)	4.56 ± 0.68	4.68 ± 0.58	0.531	4.71 ± 0.84	4.29 ± 0.85*	0.031
HDL (mmol/L)	1.28 ± 0.58	1.25 ± 0.22	0.776	1.15 ± 0.39	1.20 ± 0.36	0.636
LDL (mmol/L)	2.70 ± 0.70	2.69 ± 0.57	0.955	2.79 ± 0.79	2.40 ± 0.71*	0.032
Liver and kidney function indicators						
ALT (IU/L)	37.00 ± 28.42	37.18 ± 42.98	0.983	49.16 ± 32.36	38.52 ± 24.53	0.212
AST (IU/L)	23.42 ± 15.06	28.67 ± 20.42	0.264	33.11 ± 20.39	27.25 ± 15.86*	0.049
γ-GGT (IU/L)	34.20 ± 22.15	36.35 ± 25.39	0.803	48.53 ± 32.59	40.63 ± 47.49	0.361
BUN (mmol/L)	4.44 ± 1.00	4.38 ± 0.94	0.807	4.11 ± 1.04	3.87 ± 0.81	0.330
Cre (umol/L)	59.80 ± 7.70	59.51 ± 8.83	0.914	59.14 ± 10.05	57.71 ± 13.94	0.595
Inflammatory indicators						
CRP (mg/L)	4.62 ± 2.57	4.28 ± 2.22	0.057	4.79 ± 2.26	3.80 ± 2.33	0.105
IL-6 (pg/ml)	5.23 ± 3.34	3.96 ± 1.81	0.194	6.26 ± 6.19	3.79 ± 2.01*	0.012
TNF-α (pg/ml)	7.97 ± 2.46	8.51 ± 9.83	0.489	8.97 ± 3.68	6.20 ± 2.34	0.712
IL-8 (pg/ml)	106.75 ± 289.54	99.00 ± 267.07	0.944	178.48 ± 735.60	70.80 ± 143.67	0.326
WBC (10^9^/L)	7.12 ± 1.42	6.82 ± 1.81	0.489	6.81 ± 1.76	6.97 ± 1.69	0.712

Data are mean ± SD or n (%)

*BMI* body mass index, *WHR* waist-to-hip ratio, *F-G score* Ferriman-Gallwey score, *T* testerone, *LH* luteinizing hormone, *FSH* follicle- stimulating hormone, *DHEA-S* dehydroepiandrosterone, *SHBG* sex hormone-binding globulin, *FAI* free androgen index, *E2* estradiol, *P* progesterone, *PRL* prolactin, *HbA1c* hemoglobin A1c, *FBG* fasting blood glucose, *FINS* fasting insulin, *AUCI* area under curve of insulin, *HOMA- IR* homeostasis model assessment of insulin resistance, *TG* triglycerides, *TC* total cholesterol, *HDL* high-density lipoprotein cholesterol, *LDL* low-density lipoprotein cholesterol, *ALT* alanine transaminase, *AST* aspartate transaminase, *γ-GGT* gamma-glutamyl transpeptidase, *BUN* blood urea nitrogen, *Cre* creatinine, *CRP* C-reactive protein, *IL* interleukin, *TNF-α* tumor necrosis factor-α, *WBC* white blood cell count

^a^Amenorrhea was defined as amenorrhea lasting more than 3 previous menstrual cycles or more than 6 months

^b^Oligomenorrhoea was defined as having a menstrual cycle of 35 days or more

^c^Polycystic ovaries were defined by an antral follicle count of 12 or more or by a volume of more than 10 cm^3^ in at least 1 ovary

* *P* value < 0.05, ** *P* value < 0.01, *** *P* value < 0.001, post-treatment vs. baseline group

### Baseline and biochemical clinical data in PCOS

The baseline clinical characteristics of the two randomized treatment groups were matched. Both groups had hyperinsulinemia, and participants were overweight with visceral fat accumulation (BMI 27.75 vs 29.23 kg/m²; waist circumference 93.07 vs 95.78 cm; WHR 0.90 vs 0.91 in the CPA/EE + Met group and GLP-1RA + Met group, respectively). They also showed reproductive dysfunction, including loss of dominant follicles (0% of patients had dominant follicles in both groups), polycystic ovaries (40 vs 30%), menstrual disturbances such as amenorrhea (40 vs 30%) and oligomenorrhea (36.67 vs 43.33%), elevated blood testosterone levels, LH levels, and LH/FSH ratio. Moreover, before treatment, the percentage of participants with glucose intolerance/diabetes was 20% in the CPA/EE+Met group and 36.67% in the GLP-1RA+Met group, while nearly 78 .33 %of participants had insulin resistance (mean HOMA-IR over2 .5) [18, 19].

### Anthropometric measurements and Liver and kidney function changes

**Fig. 3:**
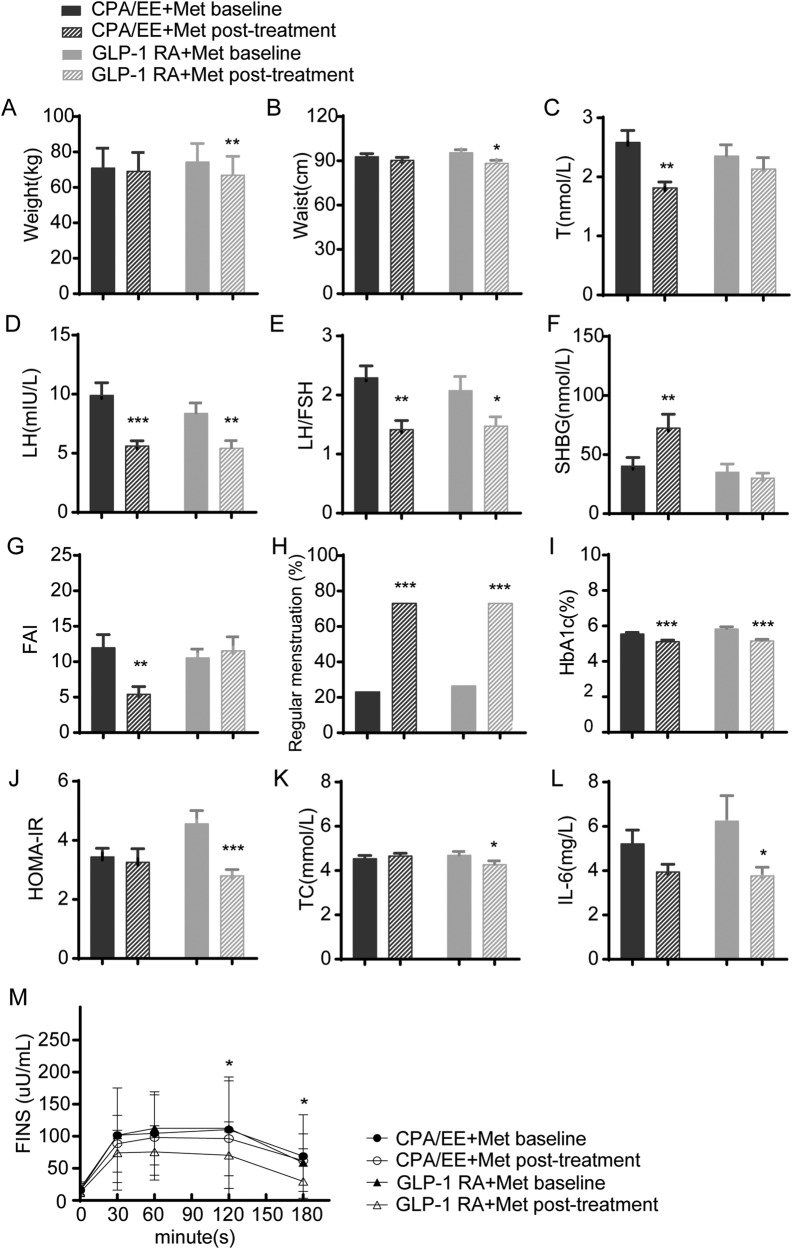
Changes in the characteristics of clinical parameters were observed after CPA/EE+Met and GLP-1RA + Met treatments. These changes included weight (**A**), Waist (**B**), T (**C**), LH (**D**), LH/FSH (**E**), SHBG (**F**), FAI (**G**), Regular menstruation (**H**), HAb1c (**I**), HOMA-IR (**J**), TC (**K**), IL-6 (**L**), FINS (**M**), *n* = 30. The data are presented as mean ± SEM. * *P* value < 0.05, ** *P* value < 0.01, *** *P* value < 0.001 (vs. before treatment in each group). Changes in FINS (**M**), after CPA/EE+Met and GLP-1 RA+Met treatment * *P* value < 0.05 (vs. before treatment in GLP-1RA + Met)

After 12 weeks of treatment, compared to CPA/EE + Met, GLP-1RA + Met treatment led to a more robust decrease in Weight (ΔWeight), BMI (ΔBMI) and Waist (ΔWaist) levels and decreased average body weight by −7.40 kg (Table 1, Table 2, Fig. 3A, B, and Supplementary Fig. 1). Compared with baseline, there was no significant difference in weight, BMI, wasit, liver and kidney function in CPA/EE + Met (*P* > 0.05). (Table 1). There were no significant differences in WHR, ALT and kidney function between the two groups after treatment.(*P* > 0.05). (Table 2). Interestingly, among these parameters, the levels of AST, and γ-GGT decreased after GLP-1RA+Met treatment but slightly increased or remained unchanged after CPA/EE + Met intervention (Supplementary Fig. 1).

**Table 2:** Characteristics change after treatment in this study

Characteristics	CPA/EE +Met (*n* = 30)	GLP-1 RA +Met (*n* = 30)	*t/z*	*P* value
Biometric features				
ΔWeight (kg)	−1.85 ± 3.25	−7.40 ± 5.91^***^	−4.312	<0.001
ΔBMI (kg/m²)	−0.76 ± 1.31	−2.90 ± 2.30^***^	−4.288	<0.001
ΔWaist (cm)	−2.53 ± 4.90	−7.22 ± 4.98^**^	3.675	0.001
ΔWHR	−0.01 ± 0.08	−0.02 ± 0.04	−0.340	0.734
Sex hormones and related indicators				
ΔF-G score	−0.53 ± 0.73	−0.37 ± 0.56	−0.849	0.396
ΔT (nmol/L)	−0.76 ± 0.85	−0.22 ± 1.37^*^	−2.196	0.028
ΔLH (mIU/ml)	−4.29 ± 6.21	−2.96 ± 4.86	−0.739	0.460
ΔFSH (mIU/ml)	0.03 ± 1.76	−0.50 ± 1.80	−0.931	0.352
ΔLH/FSH	−0.88 ± 1.15	−0.60 ± 1.38	−1.301	0.193
ΔDHEA-S (ug/dL)	−2.69 ± 89.20	−33.34 ± 136.50	1.030	0.307
ΔSHBG (nmol/L)	32.27 ± 63.52	−5.01 ± 37.81^*^	−2.055	0.040
ΔFAI	−6.55 ± 6.82	1.00 ± 8.30***	−3.855	<0.001
ΔE2 (pg/ml)	−4.20 ± 53.98	−1.30 ± 61.13	−0.917	0.359
ΔP (ng/ml)	0.10 ± 2.49	−0.21 ± 2.55	−0.893	0.372
ΔPRL (ng/ml)	−3.54 ± 9.88	−0.27 ± 20.27	−0.030	0.976
Biochemical profile of glucose metabolism				
ΔHbA1c (%)	−0.43 ± 0.53	−0.66 ± 0.46^*^	−2.217	0.027
ΔHbA1c (mmol/mol)	−4.87 ± 5.82	−7.40 ± 5.07^*^	−2.054	0.040
ΔFBG (mmol/L)	−0.38 ± 0.57	−0.54 ± 0.87	−0.673	0.501
ΔAUCI	−26.01 ± 139.14	−98.32 ± 109.77^*^	−2.366	0.018
ΔFINS (uU/ml)	0.21 ± 8.79	−6.21 ± 9.81^*^	−2.203	0.028
ΔOGTT-30 min insulin (uU/ml)	−14.38 ± 29.07	−29.07 ± 68.85	−0.111	0.912
ΔOGTT-60 min insulin (uU/ml)	−7.73 ± 55.30	−36.73 ± 50.08^*^	2.129	0.037
ΔOGTT-120 min insulin (uU/ml)	−10.41 ± 79.82	−40.83 ± 64.37	−1.575	0.115
ΔOGTT-180 min insulin (uU/ml)	−4.67 ± 75.01	−27.71 ± 38.77^*^	−2.403	0.016
ΔHOMA-IR	−0.18 ± 1.91	−1.76 ± 2.25^**^	−2.750	0.006
Biochemical profile of lipid metabolism				
ΔTG (mmol/L)	0.02 ± 0.77	−0.35 ± 0.92^**^	−2.891	0.004
ΔTC (mmol/L)	0.13 ± 0.69	−0.41 ± 0.93^*^	−2.122	0.034
ΔHDL (mmol/L)	−0.03 ± 0.52	0.05 ± 0.50	−0.237	0.813
ΔLDL (mmol/L)	−0.01 ± 0.54	−0.40 ± 1.17^*^	−1.974	0.048
Liver and kidney function indicators				
ΔALT (IU/L)	0.19 ± 28.98	−10.64 ± 33.04	−0.185	0.853
ΔAST (IU/L)	5.25 ± 14.33	−5.80 ± 26.03^*^	−2.129	0.033
Δγ-GGT (IU/L)	2.15 ± 18.92	−7.91 ± 30.07^*^	−2.269	0.023
ΔBUN (mmol/L)	−0.06 ± 1.21	−0.24 ± 1.31	−0.577	0.564
ΔCre (umol/L)	−0.29 ± 7.86	−1.44 ± 14.50	−0.651	0.515
Inflammatory indicators				
ΔCRP (mg/L)	−0.35 ± 2.19	−0.98 ± 2.72	−1.428	0.153
ΔIL-6 (pg/ml)	−1.27 ± 2.74	−2.47 ± 6.17	−0.155	0.877
ΔTNF-α (pg/ml)	0.54 ± 0.70	−2.76 ± 3.38	−1.472	0.141
ΔIL-8 (pg/ml)	−7.66 ± 394.76	−107.68 ± 735.41	−0.325	0.745
ΔWBC (10^9^/L)	−0.29 ± 1.56	0.16 ± 1.84	−0.820	0.416

Data are mean ± SD. Δ, The changes between post-treatment and baseline

^*^ *P* value < 0.05, ** *P* value < 0.01, *** *P* value < 0.001

### Reproductive function and Gonadal parameters changes

After 12 weeks of treatment, the recovery rate of menstrual cycle was 76.66% (23/30) in the CPA/EE + Met group (*P* < 0.01), and 73.3% (22/30) in the GLP-1RA+Met group (*P* < 0.01). Polycystic ovaries and amenorrhea had decreased in both groups (*P* < 0.01 or *P* < 0.05) (Table 1, Fig. 3H). Furthermore, the percentage of participants with dominant follicles increased from 0 to 20% with GLP-1RA+Met treatment. The prevalence of polycystic ovaries in the CPA/EE + Met group decreased from 40 to 0% (Table 1). Indexes of hyperandrogenemia, including T, LH, LH/FSH, SHBG, and FAI improved in the CPA/EE+ Met group while only LH improved in the GLP-1RA+Met group (Table 1, Fig. 3C-G). Compared to GLP-1 RA + Met treatment, CPA/EE + Met led to a more significant decrease in ΔT and ΔFAI levels as well as an increase in ΔSHBG level (Table 2, Supplementary Fig. 1). However, no significant differences were found between two groups on F-B score, E_2_, FSH, LH, LH/FSH and PRL (*P* > 0.05), (Table 2). CPA/EE + Met treatment resulted in a more pronounced decrease in ΔT and ΔFAI levels and an increase in ΔSHBG levels compared to GLP-1RA + Met (Table 2, supplementary Figure 1). However, there were no significant differences in F-G scores, E2, FSH, LH, LH/FSH, and PRL between the two groups (*P* > 0.05) (Table 2).

### Glucolipid metabolism and inflammatory markers changes

After 12 weeks of treatment, compared to baseline, GLP-1 RA+Met showed more robust improvement than baseline on metabolic disorders, such as glucose metabolism (e.g., decreased HbA1c, FBG、AUC-INS, FINS level, OGTT-120 min, 180 min and HOMA-IR), lipid metabolism (e.g., decreased TC and LDL), and inflammatory markers (e.g., decreased Interleukin 6 (IL-6) levels), (*P* < 0.05 or *P* < 0.01) (Table 1, Fig. 3 I-M). Compared to CPA/EE + Met, GLP-1RA + Met treatment led to a more robust decrease in ΔHbA1c, ΔAUCI and ΔFINS and ΔOGTT-60 min, ΔOGTT-180 min, ΔHOMA-IR, ΔTG, ΔTC, ΔLDL level (*P* < 0.05 or *P* < 0.01), (Table 2, Supplementary Figure 1). However, there were no significant differences on inflammatory markers between the two groups (*P* > *0.05*). (Table 2).

### Plasma proteome changes after treatment

To explore the endocrine mechanisms underlying these two interventions, we conducted mass spectrometry proteomics analysis in the plasma. Pearson correlation analysis showed high reproducibility of the proteomic assay (coefficient > 0.6), which justified further analyses (Supplementary Fig. 2A). Of 1014 identified plasma proteins that passed quality control, 182 exhibited significantly downregulated expression, and 6 demonstrated upregulated expression after treatment in the CPA/EE + Met group compared with the baseline expression levels; expression levels of 41 and 14 proteins were significantly downregulated and upregulated after GLP-1RA + Met treatment compared with the baseline levels, respectively (fold change ≥1.5 and *p* value < 0.05; Fig. 2A, B). Next, functional enrichment analysis was performed using the Gene Ontology biological process (GO BP) resource. The top enriched GO terms were selected for exhibition (Fig. 2C). Most of the significantly changed proteins after CPA/EE + Met treatment showed downregulated expression and enrichment in response to reactive oxygen species [20], platelet degranulation [21], hydrogen peroxide catabolic process, cellular oxidant detoxification [22] and immune response [23]. In addition, GLP-1RA+Met treatment led to upregulated protein expression and enrichment in cellular oxidant detoxification and oxygen transport, as well as a downregulated response to reactive oxygen species and platelet degranulation. These two treatments consistently decreased the production of plasma proteins that functioned in response to reactive oxygen species, platelet degranulation, positive regulation of NF-κB transcription factor activity and leukocyte migration. However, proteins that exhibited upregulated expression in the GLP-1RA + Met group and downregulated expression in the CPA/EE + Met group were enriched in terms related to reduced cellular oxidant detoxification and response to hydrogen peroxide (Fig. 2C).

### Validation of plasma candidate biomarkers

**Fig. 4:**
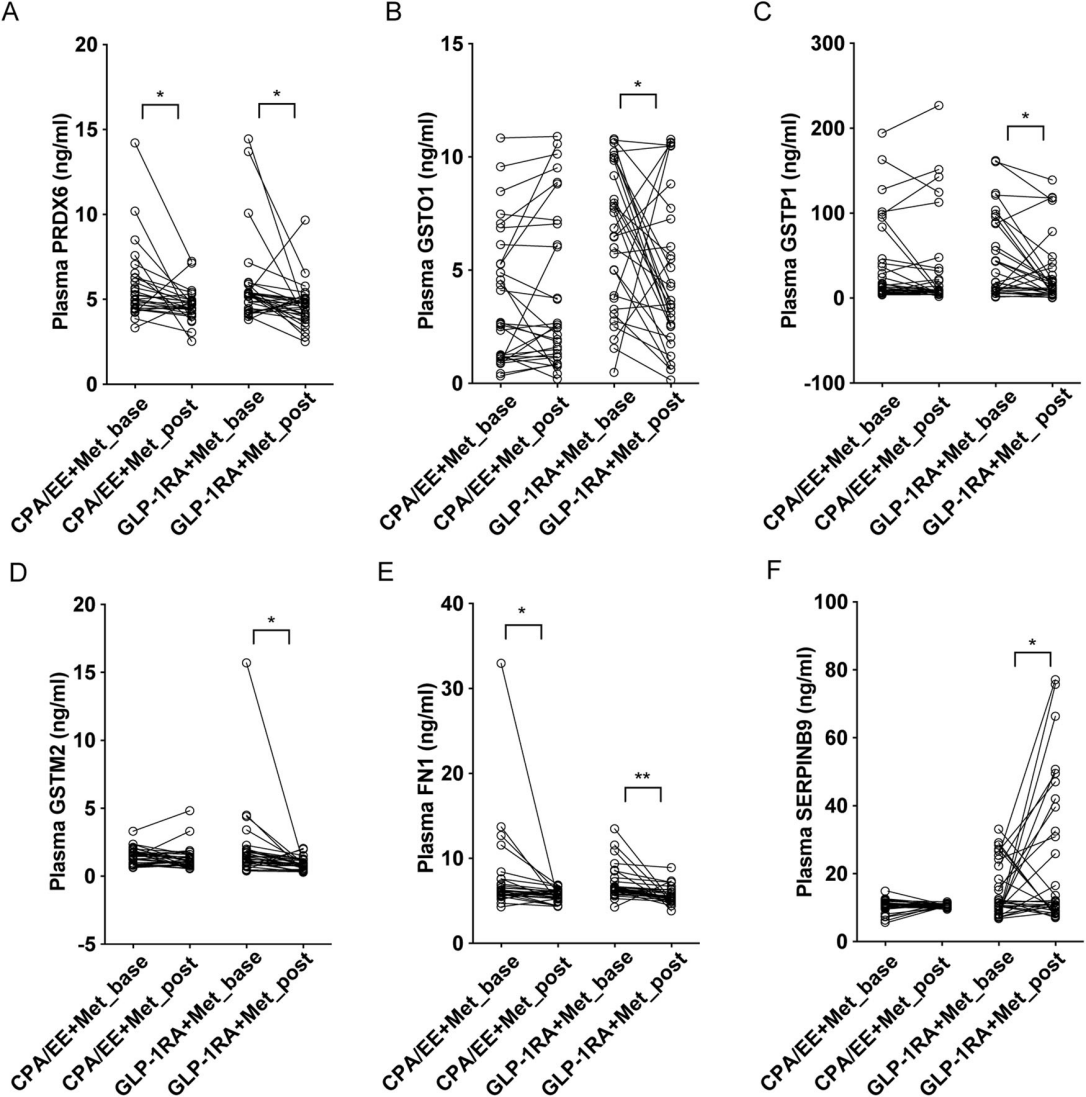
ELISA validation of plasma candidate protein marker levels. Plasma levels of PRDX6 (**A**), GSTO1 (**B**), GSTP1 (**C**), GSTM1 (**D**), FN1 (**E**) and SERPINB9 (**F**) were measured in the CPA/EE+Met and GLP-1RA+Met groups at baseline and post treatment. *n* = 27–30. * *P* value < 0.05, ** *P* value < 0.01, *** *P* value < 0.001, paired *t* test

To validate the candidate biomarkers selected by proteomic analysis, we enlarged the sample scale to 27-30 plasma samples from each group and reanalyzed the levels of the candidates PRDX6, GSTO1, GSTP1, GSTM2, FN1, and SERPINB9 via ELISA. Our results indicated that one marker for response to reactive oxygen species, PRDX6, and a platelet degranulation process marker, FN1, were significantly decreased after both treatments (Fig. 4A, E). Interestingly, it was only after GLP-1RA+Met treatment that GSTO1, GSTP1, and GSTM2, the proteins involved in reactive oxygen species detoxification, was reduced (Fig. 4B–D), while the immune response marker SERPINB9 was elevated (Fig. 4F). In summary, our findings showed that these plasma candidate markers, including PRDX6, FN1, and SERPINB9, may participate in PCOS development or drug management in response to reactive oxygen species, cellular oxidant detoxification, platelet degranulation, and immune response processes.

## Discussion

In this randomized controlled trial, our results demonstrate that both treatments are effective in recovering ovary morphology and promoting regular menstruation. We show that GLP-1RA + Met treatment is superior to CPA/EE + Met treatment in reducing weight and improving glycolipid metabolism in overweight/obese women with PCOS. Metformin greatly contributed to the common improvements in the clinical effect of both treatments, although some monotherapy studies of metformin failed to find many clinical benefits [24]. The drug synergy of combination therapy might explain these benefits.

GLP-1RA + Met is more effective in reducing weight, improving glucolipid metabolism, insulin sensitivity, and liver function. The combination therapy shows a synergistic effect. In this study, after the 12-week treatment, patients in the CPA/EE + Met group lost an average of 1.8 kg compared to 7.4 kg in the GLP-1RA + Met group. GLP-1RA + Met is also superior to CPA/EE + Met in reducing waist circumference; therefore, it may provide better control of abdominal obesity. Consistent with our result, Rui-Lin Ma et al. [25] reported an average weight loss of 3.76 kg and reduction of waist circumference after 12 weeks of exenatide combined with metformin treatment. In this short-term trial, GLP-1RA + Met significantly reduced FPG as well as HbA1c levels, AUCI values, FINS levels, and HOMA-IR index in patients with PCOS while CPA/EE+Met did not achieve the same therapeutic effect. Thus, GLP-1RA+Met therapy may be beneficial for shortening the duration of treatment. GLP-1RAs activate GLP-1 receptors on beta cells mimicking endogenous GLP-1 to stimulate glucose-dependent phase 2 insulin secretion [26]. Chuan Xing et al. [27] found that after 12 weeks of treatment with liraglutide at a dose of 1000 mg BID plus metformin at a dose of QD reduced FBG levels as well as insulin levels and HMOA-R index but did not significantly improve AUCI values among overweight PCOS patients; however our results showed significant improvement in AUCI values after a period of twelve weeks’ treatment suggesting that GLP-RAs+Met can significantly enhance beta cell function among overweight or obese PCOS patients.This may be due to the sample size, ethnic differences, and error correlation in model evaluation, resulting in different results of changes in glucose metabolism in different studies. Additionally, our study shows that GLP-1RA + Met can reduce TG and HDL-c levels in overweight PCOS patients. GLP-1RA’s potential mechanism of action in reducing systemic and tissue inflammation could be attributed to appetite suppression, weight loss, and a reduction in postprandial lipoprotein secretion. These effects may contribute to the modulation of blood lipid levels. This finding was inconsistent with previous reports, Rui-Lin Ma et al. [25] showed significant increases in serum TG, HDL-c levels after 12 weeks of treatment for exenatide and metformin for overweight women with polycystic ovary syndrome. This difference was also likely caused by the administration of Diane-35, as Diane-35 has been reported to increase blood lipid levels with increasing number of therapy cycles in patients with PCOS [28].

Surprisingly, GLP-1RA + Met treatment resulted in the increased occurrence of dominant follicles without androgen decrease, which might result from improved hyperinsulinemia and then restore the regular menstrual cycle and promote the formation of dominant follicles [29]. Conversely, CPA/EE + Met was more effective in reducing androgen and increasing SHBG levels than GLP-1RA + Met. Consistent with our results, previous studies reported that oral contraceptives or metformin monotherapy improved androgen and SHBG levels in PCOS women by sex-related hormone feedback or enhanced insulin sensitivity [30]. However, insulin resistance and compensatory hyperinsulinemia are present in approximately 25% of adolescents with PCOS and in 50–70% of adults, based on clinical studies [31, 32] which may also be one of the reasons for the slight decrease in androgen after treatment in our study, and it also suggests that anti-androgen therapy might not be the main therapeutic target in this kind of population.

Thus, considering the long-term benefits of improving metabolism and the urgency of pregnancy, GLP-1RA + Met is more effective than CPA/EE + Met in promoting ovulation and improving metabolic disturbance for overweight women with PCOS. Moreover, these beneficial effects also suggested the possibility of reducing the occurrence of metabolic and reproductive endocrinology disorders in PCOS patients and their future generations.

Previous studies have shown that the abundance of some plasma proteins changed significantly in PCOS patients, which contributes to the pathophysiological mechanism [33, 34] Our proteomic results indicated that both interventions led to plasma protein changes, which is mainly related to the response to reactive oxygen species, platelet degranulation. Consistently, ELISA validated that the levels of the indicators of response to reactive oxygen species (PRDX6, GSTO1, GSTP1, GSTM2) and platelet degranulation (FN1) were decreased. CPA/EE + Met treatment changed PRDX6 and FN1 levels. These interesting changes in plasma protein levels might explain the clinical outcome characteristics of both interventions to some degree.

Although a direct causal relationship could not be determined in our study, there was a correlation between the changes in plasma protein levels caused by drug intervention and the improvement of clinical symptoms. In detail, oxidative stress injury and excessive self-protection have been proven to be involved in the occurrence and development of metabolic disorders and PCOS [35]. As the key proteins of the oxidative stress process that maintain intracellular homeostasis and regulate redox signaling [36], PRDX6, GSTO1, GSTP1, and GSTM2 significantly decreased or showed a decreasing tendency after both treatments. The decrease in these protein levels could be an outcome of the improvement of metabolic disorders, but it may also contribute to improving metabolic profiles. A hypercoagulable state is associated with PCOS [37]. A recent study reported increased circulating FN1 protein levels in PCOS patient [38] and increased FN1 mRNA levels in the ovarian tissue of a PCOS mouse model [39]. Thus, it is reasonable that both treatments significantly decreased the FN1 protein level in our study. In line with our results, metformin and GLP-1RA has been found to attenuate platelet aggregation or thrombosis in mice [40, 41] These data suggest that the coagulation process contributed to the pathogenesis of PCOS and that decreased FN1 levels helped improve the PCOS process. Additionally, as a member of the serine proteinase inhibitor (serpin) family, SERPINB9 is mainly originally expressed by T lymphocytes and effectively inhibits human granzyme B (GZB) activation [42, 43]. It has been reported that GZB serum levels are increased in adolescent PCOS patients [39] and play a potential role in the development of IR by promoting the release of proinflammatory cytokines as cytotoxic molecules [44]. Our results first found significantly increased circulating SERPINB9 protein levels only with GLP-1RA + Met treatment in overweight/obese PCOS patients, and GLP-1RA + Met treatment robustly induced improvements in systemic metabolism and ovulation in PCOS women. However, whether increased SERPINB9 levels contributed to the effect of treatment needs to be identified. In short, circulating proteins such as PRDX6, FN1 and SERPINB9 provide further clues for exploring the effective molecular mechanism of treatment and might be reference candidate biomarkers for assisting with choosing the most suitable drugs for overweight/obese PCOS patients.

In addition to the proteomics analysis that provides new molecular insights, another major strength of the current study is the comprehensive phenotypic characterization across a timeline. Limitations of this trial include a relatively small sample size and single-center design. Additionally, lifestyle changes were not controlled for as we aimed to isolate the effects of the drug on overweight patients with PCOS. Given the special administration method (I.H.) with a GLP-1RA, participants and investigators were not blinded to the treatment in this trial. Future studies can test newer GLP-1RAs associated with a lower incidence of gastrointestinal side effects, and the prospective studies should be designed to verify the specific role of plasma candidate biomarkers we found in the treatment of PCOS. The adverse effects of GLP-1RA+Met were mainly observed in the gastrointestinal system, which might be related to its action in the autonomic nervous system [45]. A small number of patients treated with CPA/EE + Met exhibited weight gain, irregular menstrual bleeding, and impaired liver functions, which calls for caution for this therapy regimen.

## Conclusion

In summary, both CPA/EE + Met or GLP-1RA + Met improved menstrual cycles and polycystic ovaries. For overweight patients with PCOS, GLP-1RA + Met treatment is more effective than CPA/EE + Met treatment at improving metabolic disorders while also having obvious advantages at increasing ovulation rates among patients. Therefore we recommend that GLP-1RA + Met should be used for patients suffering from severe combined glycolipid metabolism disorders along with high body fat percentages as it can help correct metabolic disorders quickly while also improving menstruation and ovulation. Plasma proteomics analyses shed light on potential molecular mechanisms and suggested several candidate biomarkers, such as PRDX6, FN1, and SERPINB9, for the clinical prevention, diagnosis, or management of overweight PCOS patients. These results present a clinical advance for treating PCOS.

### Supplementary information

Supplementary Figure 1

Supplementary Figure 2

Supplementary Figuer Legends

Supplementary Table 1

Supplementary Table 2

## Data Availability

The datasets generated during and/or analyzed during the current study are available from the corresponding author on reasonable request.
