# Supplementary figures and images for: Effects and plasma proteomic analysis of GLP-1RA versus CPA/EE, in combination with metformin, on overweight PCOS women: a randomized controlled trial
Source: Endocrine. 2023 Aug 31;83(1):227–41. doi: 10.1007/s12020-023-03487-4 (PMC10806039; doi:10.1007/s12020-023-03487-4)

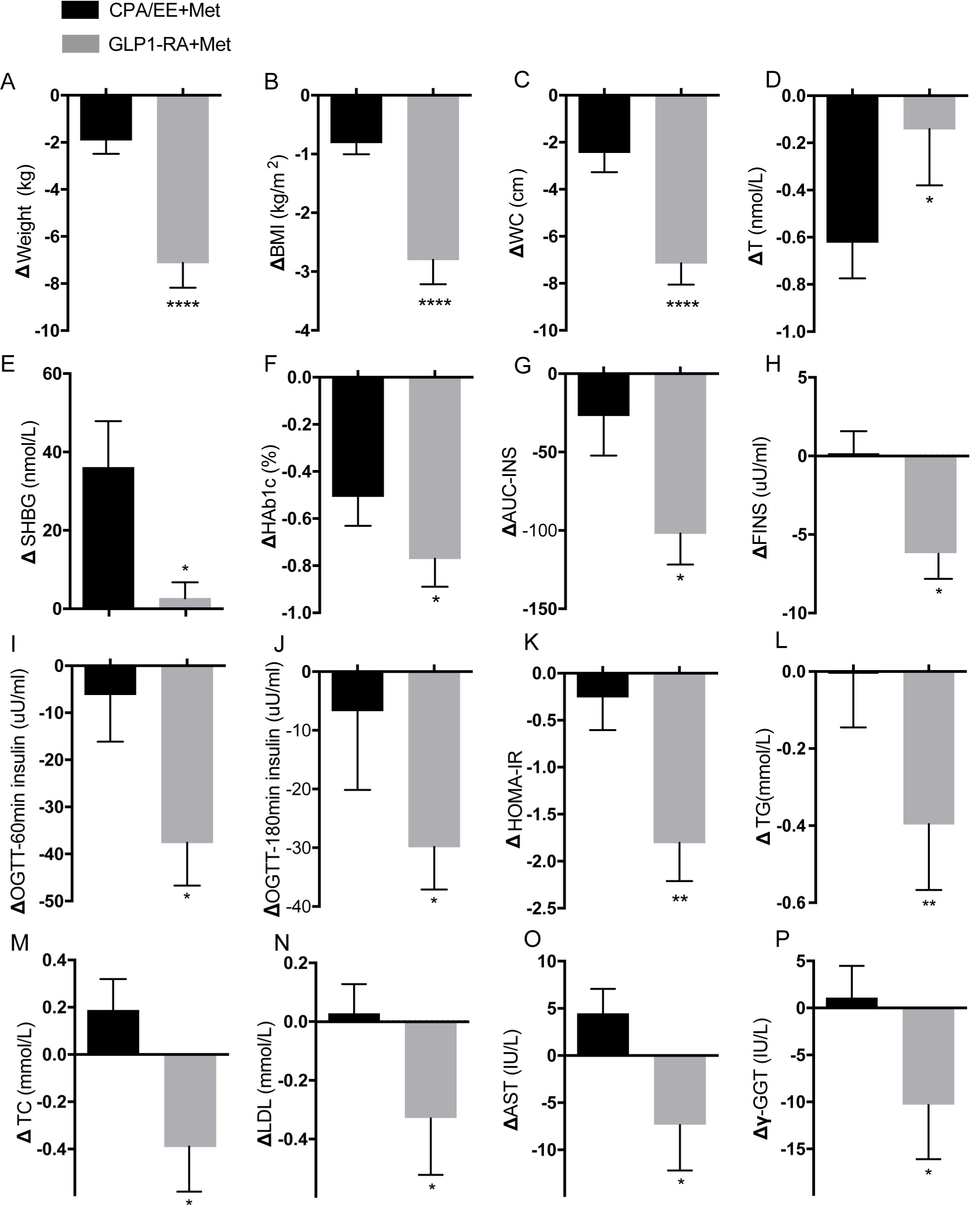

Supplement: Supplementary file 1 — Supplementary Figure 1 [file 12020_2023_3487_MOESM1_ESM.tif]

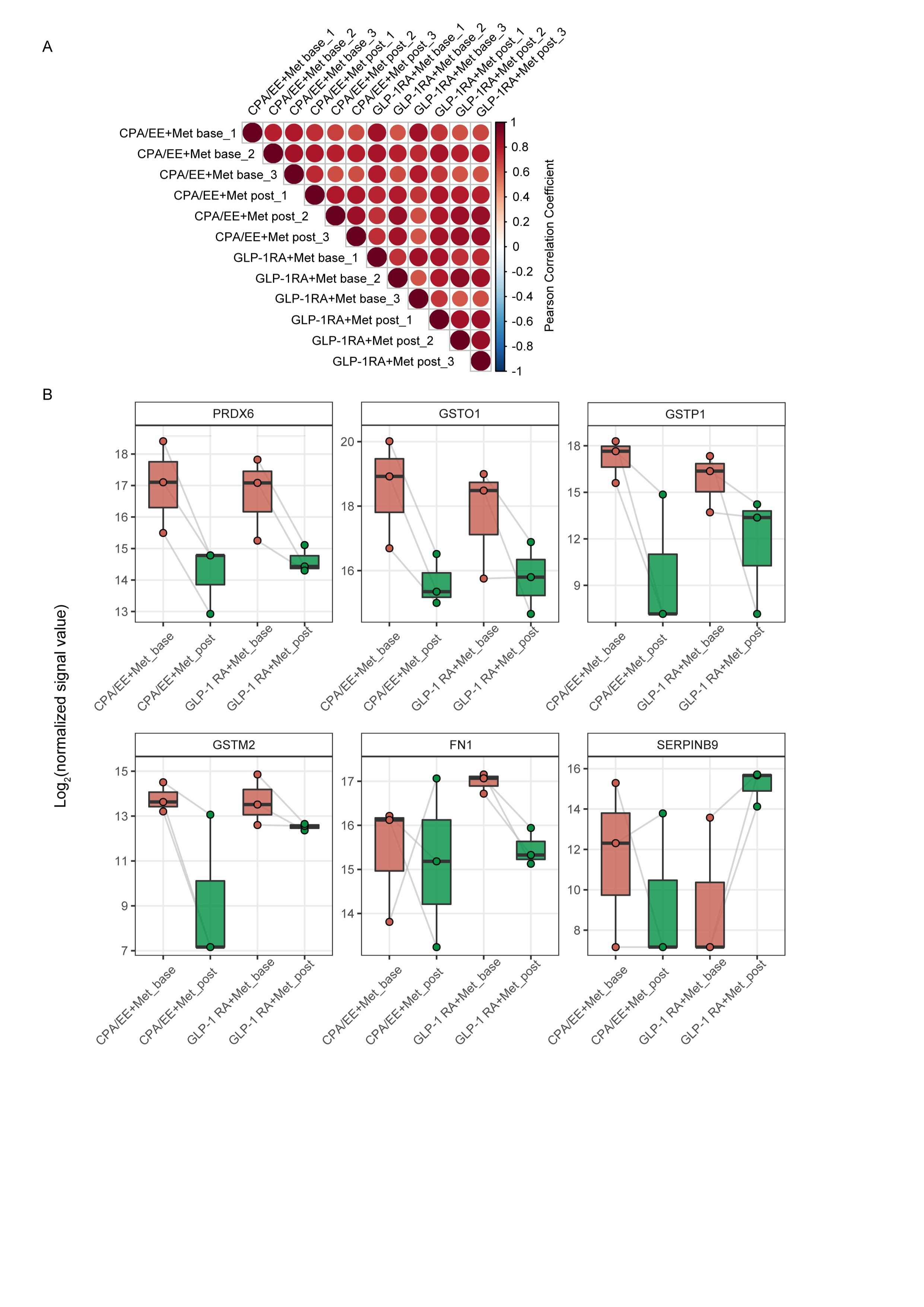

Supplement: Supplementary file 2 — Supplementary Figure 2 [file 12020_2023_3487_MOESM2_ESM.tif]
