# Supplementary material for: Effects and plasma proteomic analysis of GLP-1RA versus CPA/EE, in combination with metformin, on overweight PCOS women: a randomized controlled trial
Source: Endocrine. 2023 Aug 31;83(1):227–41. doi: 10.1007/s12020-023-03487-4 (PMC10806039; doi:10.1007/s12020-023-03487-4)
Supplement: Supplementary file 3 — Supplementary Figuer Legends [file 12020_2023_3487_MOESM3_ESM.docx]

Supplementary Figure 1. Significant changes in clinical parameters were compared between the two treatment groups. Change values of BMI (A), weight (B), waist circumference (C), testosterone (D), SHBG (E), FAI (F), HAb1c (G), AUC-INS (H), FINS (I), OGTT 60 min insulin (J), OGTT 180 min insulin (K), HOMA-IR (L), TG (M), TC (N), LDL (O), AST (P) and γ-GGT level (Q) comparison between CPA/EE+Met and GLP-1 RA+Met treatment, n=30. Data are presented as the mean ± SEM, Mann-Whitney test, * P value < 0.05, ** P value < 0.01, **** P value < 0.0001.

Supplementary Figure 2. Reproducibility analysis of the proteomic data and normalized expression levels of six candidate proteins. (A) Pearson correlations of the proteomic data among samples. (B) Boxplot of normalized signal values of six candidate proteins, moderated paired t-test by limma.
