# Supplementary material for: Effects and plasma proteomic analysis of GLP-1RA versus CPA/EE, in combination with metformin, on overweight PCOS women: a randomized controlled trial
Source: Endocrine. 2023 Aug 31;83(1):227–41. doi: 10.1007/s12020-023-03487-4 (PMC10806039; doi:10.1007/s12020-023-03487-4)
Supplement: Supplementary file 4 — Supplementary Table 1 [file 12020_2023_3487_MOESM4_ESM.docx]

Supplementary Table 1. Adverse effects of participants during the trial.

|  | CPA/EE+Met (n=30) | GLP-1 RA+Met (n=30) |
| --- | --- | --- |
|  | no. of women/total no. (%) | |
| Weight gain | 4/30(13.33) | 0/30 |
| Irregular menstrual bleeding | 2/30(6.67) | 0/30 |
| Aminopherase elevation | 1/30(3.33) | 0/30 |
| Diarrhea | 0/30 | 4/30(13.33) |
| Nausea | 0/30 | 4/30(13.33) |
| Vomiting | 0/30 | 4/30(13.33) |
| Indigestion | 0/30 | 2/30(6.67) |
