# Supplementary material for: Effects and plasma proteomic analysis of GLP-1RA versus CPA/EE, in combination with metformin, on overweight PCOS women: a randomized controlled trial
Source: Endocrine. 2023 Aug 31;83(1):227–41. doi: 10.1007/s12020-023-03487-4 (PMC10806039; doi:10.1007/s12020-023-03487-4)
Supplement: Supplementary file 5 — Supplementary Table 2 [file 12020_2023_3487_MOESM5_ESM.docx]

Supplementary 2. The reference ranges and test information for clinical parameters.

| Indicators | Reference range | Test method | Instrument model | Manufacturers |
| --- | --- | --- | --- | --- |
| T (nmol/L) | 0.38-1.97nmol/L | chemiluminescence immunoassay | Automatic chemiluminescence immunoanalyzer, Abbott i 2000 | China Abbott Laboratories |
| LH (mIU/ml) | Follicular phase: 2.39-6.6；ovulation phase: 9.06-72.24；luteal phase: 0.90-9.33；after menopause: 10.39-64.57 |  |  |  |
| FSH (mIU/ml) | Follicular phase: 3.03-8.08；ovulation phase: 2.55-16.69；luteal phase: 1.38-5.47；after menopause: 26.72-133.41 |  |  |  |
| LH/FSH | ＜2 | Calculation | - | - |
| DHEA-S (ug/dL) | 238.4-539.3ug/dL | chemiluminescence immunoassay | Automatic chemiluminescence immunoanalyzer, Abbott i 2000 | China Abbott Laboratories |
| SHBG (nmol/L) | 34.3-147.7nmol/L |  |  |  |
| FAI |  |  |  |  |
| E2 (pg/ml) | Follicular phase: 21-251；ovulation phase: 38-649；luteal phase: 21-312；after menopause: ＜10-144 |  |  |  |
| P (ng/ml) | Follicular phase: ＜0.1-0.3；luteal phase: 1.2-15.9；after menopause: ＜0.1-0.2 |  |  |  |
| PRL (ng/ml) | 5.18-26.53 |  |  |  |
| HbA1c (%) | 4-6% | anion-exchange high-performance liquid chromatography. | Automatic hemoglobin A1c analyzer, G8-90SL | TOSH CORPORATION |
| HbA1c (mmol/mol) | 20-42mmol/mol | Calculation | - | - |
| FBG (mmol/L) | 3.9-6.1mmol/L | hexokinase method | Automatic biochemical analyzer, AU5800-2-2 | Beckmann Kurt Co. LTD, USA |
| AUCI | AUCI=(0+2×I30+3×I60+4×I120+2×I180)/4 | Calculation | - | - |
| FINS (uU/ml) | 1.5-15uU/ml | chemiluminescence immunoassay | Automatic chemiluminescence immunoanalyzer, Abbott i 2000 | China Abbott Laboratories |
| OGTT-30min insulin (uU/ml) | 25-85uU/ml |  |  |  |
| OGTT-60min insulin (uU/ml) | 15-50uU/ml |  |  |  |
| OGTT-120min insulin (uU/ml) | 15-40uU/ml |  |  |  |
| OGTT-180min insulin (uU/ml) | 5-17uU/ml |  |  |  |
| HOMA-IR | HOMA-IR=FINS*FBG/22.5 | Calculation | - | - |
| TG (mmol/L) | 0-1.7mmol/L | Colorimetric method | Automatic biochemical analyzer, AU5800-2-2 | Beckmann Kurt Co. LTD, USA |
| TC (mmol/L) | 0-5.2mmol/L |  |  |  |
| HDL (mmol/L) | 0.9-1.68mmol/L |  |  |  |
| LDL (mmol/L) | 2.07-3.1mmol/L |  |  |  |
| ALT (IU/L) | 7-40IU/L | Colorimetric method | Automatic biochemical analyzer, AU5800-2-2 | Beckmann Kurt Co. LTD, USA |
| AST (IU/L) | 13-35IU/L |  |  |  |
| γ-GGT (IU/L) | 7-45IU/L |  |  |  |
| BUN (mmol/L) | 2.9-8.2mmol/L |  |  |  |
| CREA (umol/L) | 45-105umol/L |  |  |  |
| CRP (mg/L) | 0-8mg/L | IMMULITE 1000 Immunoassay System | Chemiluminescence immunoanalyzer | (Siemens Healthcare Diagnostics Inc |
| IL-6 (pg/ml) | 0-3.4pg/ml |  |  |  |
| TNF-α (pg/ml) | 0-8.1pg/ml |  |  |  |
| IL-8 (pg/ml) | ＜62pg/ml |  |  |  |
| WBC (10^9/L) | 3.5-9.5 | fluorescent staining | Automatic modular blood and body fluid analyzer | Sysmex, Japan |
